# Factors Influencing Abortion Decision-Making Processes among Young Women

**DOI:** 10.3390/ijerph15020329

**Published:** 2018-02-13

**Authors:** Mónica Frederico, Kristien Michielsen, Carlos Arnaldo, Peter Decat

**Affiliations:** 1 International Centre for Reproductive Health (ICRH), Ghent University, 9000 Gent, Belgium; kristien.michielsen@ugent.be; 2 Centro de Estudos Africanos, Universidade Eduardo Mondlane, C. P. 1993, Maputo, Mozambique; carlos.arnaldo@uem.ac.mz; 3 Department of Family Medicine and primary health care, Ghent University, 9000 Gent, Belgium; Peter.decat@ugent.be

**Keywords:** abortion, decision-making, young women, Maputo, Quelimane

## Abstract

*Background:* Decision-making about if and how to terminate a pregnancy is a dilemma for young women experiencing an unwanted pregnancy. Those women are subject to sociocultural and economic barriers that limit their autonomy and make them vulnerable to pressures that influence or force decisions about abortion. *Objective*: The objective of this study was to explore the individual, interpersonal and environmental factors behind the abortion decision-making process among young Mozambican women. *Methods*: A qualitative study was conducted in Maputo and Quelimane. Participants were identified during a cross-sectional survey with women in the reproductive age (15–49). In total, 14 women aged 15 to 24 who had had an abortion participated in in-depth interviews. A thematic analysis was used. *Results*: The study found determinants at different levels, including the low degree of autonomy for women, the limited availability of health facilities providing abortion services and a lack of patient-centeredness of health services. *Conclusions*: Based on the results of the study, the authors suggest strategies to increase knowledge of abortion rights and services and to improve the quality and accessibility of abortion services in Mozambique.

## 1. Introduction

Abortion among adolescents and youth is a major public health issue, especially in developing countries. Estimates indicate that 2.2 million unplanned pregnancies and 25% (2.5 million) unsafe abortions occur each year, in sub-Saharan Africa, among adolescents [1]. In 2008, of the 43.8 million induced abortions, 21.6 million were estimated to be unsafe, and nearly all of them (98%) took place in developing countries, with 41% (8.7 million) being performed on women aged 15 to 24 [2].

The consequences of abortion, especially unsafe abortion, are well documented and include physical complications (e.g., sepsis, hemorrhage, genital trauma), and even death [3,4,5,6]. The physical complications are more severe among adolescents than older women and increase the risk of morbidity and mortality [6,7]. However, the detrimental effects of unsafe abortion are not limited to the individual but also affect the entire healthcare system, with the treatment of complications consuming a significant share of resources (e.g., including hospital beds, blood supply, drugs) [5,8]. 

The decision if and how to terminate a pregnancy is influenced by a variety of factors at different levels [9]. At the individual level these factors include: their marital status, whether they were the victim of rape or incest [10,11], their economic independence and their education level [10,12]. Interpersonally factors include support from one’s partner and parental support [12]. Societal determinants include social norms, religion [9,13], the stigma of premarital and extra-marital sex [14], adolescents’ status, and autonomy within society [12]. At the organizational level, the existence of sex education [10,14], the health care system, and abortion laws influence the decisions if and where to have an abortion.

Those factors are related to power and (gender) inequalities. They limit young women’s autonomy and make them vulnerable to pressure. Additionally, the situation is exacerbated when there is a lack of clarity and information on abortion status, despite the existence of a progressive law in this regard.

For example, Mozambican law has allowed abortion if the woman’s health is at risk since the 1980s [15,16,17,18]. In 2014, a new abortion law was established that broadened the scope of the original law: women are now also allowed to terminate their pregnancy: (1) if they requested it and it is performed during the first 12 weeks; (2) in the first 16 weeks if it was the result of rape or incest, or (3) in the first 24 weeks if the mother’s physical or mental health was in danger or in cases of fetus disease or anomaly. Women younger than 16 or psychically incapable of deciding need parental consent [19,20].

Notwithstanding the progressive abortion laws in Mozambique, hospital-based studies report that unsafe abortion remains one of the main causes of maternal death in Mozambique [3]. However, hospital cases are only a small share of unsafe abortions in the country. Many women undergo an abortion in illegal and unsafe circumstances for a variety of reasons [3], such as legal restrictions, the fear of stigma [21,22,23], and a lack of knowledge of the availability of abortion services [3,9,23].

According to the 2011 Mozambican Demographic Health Survey (DHS), at least 4.5% of all adolescents reported having terminated a pregnancy [24]. Unpublished data from the records of Mozambican Association for Family Development (AMODEFA) which has a clinic that offers sexual and reproductive health services, including safe abortion, indicate that from 2010 to 2016 a total of 70,895 women had an induced abortion in this clinic, of which 43% were aged 15 to 24. Of the 1500 women that had an induced abortion in the AMODEFA clinic in the first three months of 2017, 27.9% were also in this age group [25]. These data show the high demand for (safe) abortion among young women.

For all this described above, Mozambique is an interesting place to study this decision-making process; given the changing legal framework, women may have to navigate gray areas in terms of legality, safety, and access when seeking abortion, which is stigmatized but necessary for the health, well-being, and social position of many young women.

The objective of this study is to explore the individual, interpersonal and environmental factors behind the abortion decision-making process. This entails both the decision to have an abortion and the decision on how to have the abortion. By examining fourteen stories of young women with an episode of induced abortion, we contribute to the documentation of the circumstances around the abortion decision making, and also to inform the policymakers on complexity of this issue for, which in turn can contribute to improve the strategies designed to reduce the cases of maternal morbidity and mortality in Mozambique.

## 2. Materials and Methods

This is an exploratory study using in-depth interview to explore factors related to abortion decision-making in a changing context. As research on this topic is limited, we opted for a qualitative research framework that aims to identify factors influencing this decision-making process.

### 2.1. Location of the Study

The study was conducted in two Mozambican cities, Maputo and Quelimane. These cities were selected because they registered more abortions than other cities in the same region. According to the 2014 data from the Direcção Nacional de Planificação, 629 and 698 women, respectively, were admitted to the hospital due to induced abortion complications in Maputo and Quelimane [26]. Furthermore, the two differ radically in terms of culture, with Maputo in the South being patrilineal and Quelimane in the Central Region matrilineal, which could influence the abortion decision-making process. The fieldwork took place between July–August 2016 and January–February 2017.

### 2.2. Data Collection

**Figure 1:**
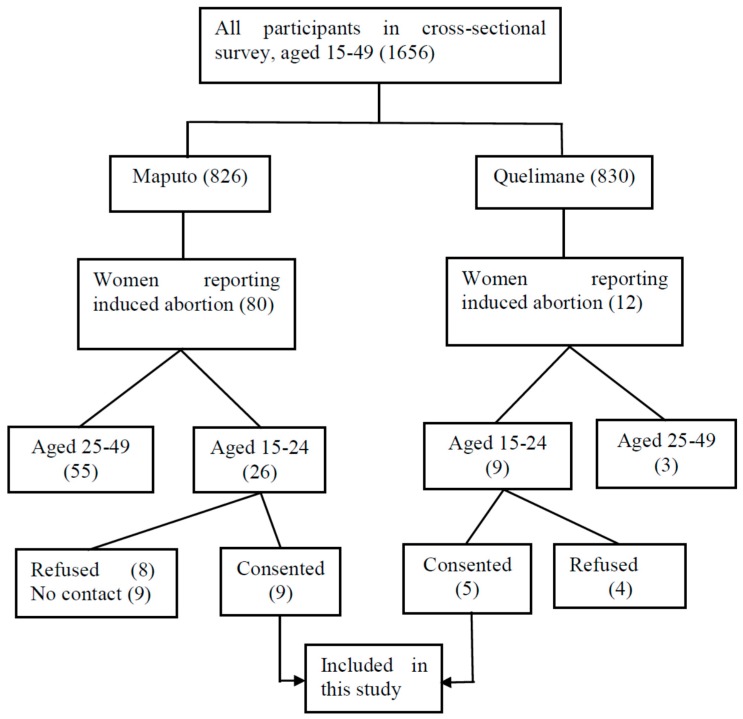
The process of recruitment of the participants.

The data were collected through in-depth interviews, asking participants about their experiences with induced abortion and what motivated them to get an abortion. To approach and recruit participants (Figure 1), we used the information collected during a cross-sectional survey with women in the reproductive age (15–49), These women were selected randomly applying multistage cluster based on household registers. The survey was designed to understand women’s sexual and reproductive health and included filter questions that allowed us to identify participants who had undergone an abortion. The information sheet and informed consent form for this household survey included information about a possible follow-up study.

Participants who were within the age-range 15–24 years and who reported having had an abortion were contacted by phone. In this contact, the researcher (MF) introduced herself, reminded the participant of the study she took part in, explained the follow-up study and asked whether she was willing to participate in this. If she did, an appointment was made at a convenient location. Before each interview, we explained to each participant why she was invited to the second interview. Participants were also informed of interview procedures, confidentiality and anonymity in the management of the data, and the possibility to withdraw from the interview at any time. In total 14, young women (15–24) agreed to participate: nine in Maputo and five in Quelimane. Six of them were interviewed twice to explore further aspects that remained unclear after the first interview. The interviews were conducted in Portuguese.

To start the interview, the participant was invited to tell her life history from puberty until the moment when the abortion occurred. During the conversation, we used probing questions to elicit more details. Gradually, we added questions related to the abortion and factors that influenced the decision process. The main questions were related to the pregnancy history, abortion decision-making, and help-seeking behaviour. The guideline was adapted from WHO tools [27,28]. Before the implementation of the guideline, it was discussed first with another Mozambican researcher to see how they fell regarding the question. After those questions were revised or removed from the guideline.

### 2.3. Data Analysis

The analysis consisted of three steps: transcription, reading, and codification with NVivo version 11(QSR International Pty Ltd., Doncaster, Australia). After an initial reading, one of the authors (MF) developed a coding tree on factors determining the decision-making. A structured thematic analysis was used to make inferences and elicit key emerging themes from the text-based data [29,30]. The coding tree was based on the ecological model, which is a comprehensive framework that emphasizes the interaction between, and interdependence of factors within and across all levels of a health problem since it considers that the behaviour affects and is affected by multiple levels of influence [31,32].

Next, the codes and the classification were discussed among the researchers (Mónica Frederico, Kristien Michielsen, Carlos Arnaldo and Peter Decat). Finally, the data was interpreted, and conclusions were drawn [33].

### 2.4. Ethical Consideration

Before the implementation of this research, we obtained ethical approval from the Institutional Committee of the Faculty of Medicine and Nacional Bioethical Committee for Health (IRB00002657). We also asked for the institutional approval of the Minister of Health and authorities at the provincial and community levels. The participants gave their informed consent after the objectives and interview procedures had been explained to them. The participants were informed that they might be contacted and invited, within six months, to participate in another interview.

### 2.5. Concepts

The providers are the people who carried out the abortion procedure. These may be categorized into skilled and unskilled providers: the former refers to a professional (i.e., nurse or doctor) offering abortion services to a client, while the latter is someone without any medical training. Another concept that requires further explanation is the legal procedure. This corresponds to a set of steps to be followed to comply with the law [19,20]. Specifically, this means that a committee should authorize the induced abortion and an identification document should be available, as well as an informed consent form from the pregnant woman. If the woman is a minor, consent is given by her legal guardian. An ultrasound exam is required to determine the gestational age.

## 3. Results

### 3.1. Characteristics of the Participants

The characteristics of the interviewees are summarized in Table 1. The 14 participants were aged 17 to 24 years. Eight had completed secondary school, four had achieved the second level of primary school, and two were university students. Almost all (13) were Christian. Five participants were studying, eight were unemployed, and one was working. The median age of their first sexual intercourse was 15.5 years. Participants reported living with one or both parents (12), with their uncle (1) or alone (1). They lived in suburban areas of Maputo and Quelimane, which are slums with poor living conditions. In these areas, most households earn their income through small businesses that also involve child labour (e.g., selling food or drinks).

Among the participants, five reported more than one pregnancy. One interviewee first had a stillbirth and then two abortions. Another woman gave birth to a girl and afterward terminated two pregnancies. Two interviewees reported two pregnancies, the first of which was brought to full term and the second one terminated. One woman first had an abortion and afterward gave birth to a child. In short, 14 interviewees in total reported on the experiences and decision-making of 16 abortions. One participant stated that the pregnancy was the consequence of rape. Of the 16 reported abortions, seven were performed after the new law came into force at the end of 2014, and nine were carried out before this time. 

### 3.2. Abortions Stories

In this study, 12 abortions were done by skilled providers and two by unskilled providers. The unskilled providers were a mother and a husband, respectively. None of the cases, whose abortion was done by a skilled provider, included in this study followed the legal procedure.

In the analysis of the interviews, we studied the personal, interpersonal and environmental factors that influenced six different types of abortion stories, see Table 2: (1) an abortion was performed because the pregnancy was unwanted; (2) an abortion was carried out although the pregnancy was wanted; (3) the abortion was done by an unskilled provider at home; (4) an abortion was carried out by a skilled provider outside the hospital; (5) a particular abortion procedure (medical or chirurgical) was chosen, and (6) the legal procedure was not followed in the hospital. Factors influencing the choice for a particular technical procedure were also examined.

### 3.3. Abortion Following an Unwanted Pregnancy

In the stories about unwanted pregnancies, mostly personal factors were mentioned as reasons, with some interviewees stating that they felt unable to be a mother at the time of the pregnancy: “*(It) was at the time that I was taking pills that I got pregnant, and I induced abortion because I was not prepared (for motherhood).*” (24 years) 

Some had had a bad experience in the past: “*Maybe I would be abandoned and it would be the same. (Sigh)... I learned with my first pregnancy.*” (23 years)

Also, the existence of another child was mentioned as a reason to have an abortion: “*I got pregnant when I was 20, and I had a baby. When I became pregnant again, my daughter was a child, and I could not have another child.*” (23 years)

For other participants, studies were the main reason why the pregnancy was not wanted: “*He was informed about it, and he said that I should keep it. However, as I wanted to continue my studies, I told him no, no (I) do not.*” (17 years)

At the interpersonal level, a lack of support from the partner was often mentioned as a reason for not wanting the baby: “*He said that he recognizes the paternity, but it is not to keep that pregnancy.*” (22 years) 

Women frequently mentioned environmental circumstances related to their poor socio-economic situation: “*I am staying at Mom's house; it is not okay to still be having* babies there.” (23 years)

“*At home, we do not have any resources to take care of this child!*” (20 years)

### 3.4. Abortion Following a Wanted Pregnancy

In these cases, the decision to abort the pregnancy was not made by the woman herself but imposed by others or by the circumstances. 

Some participants reported that their parents/family had decided what had to be done: “*They decided while I was at school. If (it) was my decision I would keep it because I wanted it.*” (18 years). 

Other young women indicated the refusal of paternity as a reason to terminate the pregnancy.

“*Because my son’s father did not accept the (second) pregnancy. There was a time, we argued with each other, and we terminated the relationship. Later, we started dating again, and I got pregnant. He said it was not possible.*” (21 years)

“*(he) impregnated me and after that, he dumped me, (smiles)… I went to him, and I said that I was pregnant. He said eee: I do not know, that is not my child.*” (20 years).

Some women told the interviewers that they were convinced by their boyfriend to have an abortion: “*I talked to him, and he said okay we are going to have an abortion and I accepted.*” (22 years)

Others mentioned their partner’s indecision and changing attitude as a reason to get an abortion, even though they did want the baby:

“*I told him I was pregnant. First, he said to keep it. (Next) He was different. Sometimes he was calling me, and other times not. I understood that he did not want me.*” (20 years)

The fear of being excluded from their family due to their pregnancy was another reason reported by participants: “*So I went to talk with my older sister, and she said eee, you must abort because daddy will kick you out of our home.*” (20 years)

“*As I am an orphan, and I live with my uncle, they were going to kick me out. No one would assist me.*” (20 years)

### 3.5. Location of the Abortion: Home-Based Versus Hospital-Based

Two young women reported having had the abortion at home by an unskilled provider. It seems that these unskilled providers than the women (i.e. family members, partner) made the decisions. 

“*It was mammy and my sister (who provided the induced abortion services). My sister knows these things.*” (18 years)

“*He (the father of the child) came to my house and took me back to his house. It was that moment when I aborted.*” (21 years)

Of the 16 abortions, seven were performed through health services, by a skilled provider. For some of them, the choice for a health service was influenced by the fact of knowing someone at the health facility. 

“*I went to talk to her (friend), and she said that “I have an aunt who works at the hospital, she can help you. Just take money”.*” (20 years)

“*I Already knew who could induce it (abortion). No, I knew that person. I went to the hospital, and I talked to her, (and) she helped me.*” (22 years)

Other participants went to the health facility, but due to the lack of money to pay for an abortion at the facilities they sought help out of the health facility: “*They charged us money that we did not have. The ladies did not want to negotiate anything. I think they wanted 1200 mt (17.1 euros) if I am not wrong. He had a job, but he (boyfriend) did not have that amount of money.*” (22 years) 

Some participants reported that they had an abortion outside regular facilities because the health provider recommended going to his house: “*She (mother) was the one who accompanied me. She is the one who knows the doctor. We went to the central hospital, but he (the doctor) was very busy, and he told us to go to his house.*” (17 years)

Others reported the fear of signing a document as a reason to seek help outside of official channels: “*I heard that to induce abortion at the hospital it is necessary for an adult to sign a consent form. I was afraid because I did not know who could accompany me. Because at that time I only wanted to hide it from others.*” (22 years).

### 3.6. Abortion Procedure 

The women were not able to explain why a particular abortion procedure (i.e., pills or aspiration, curettage) was used. It appears that they were not given the opportunity to choose and that they submitted themselves to the procedure proposed by the provider. 

“*The abortion was done here at home. They just went to the pharmacy, bought pills and gave them to me.*” (18 years)

### 3.7. Legal Procedure

None of those treated at the hospital stated that legal procedures were followed. They also mentioned that they had to pay without receiving any official receipt.

“*First we got there and talked to a servant (a helper of the hospital). The servant asked for money for a refreshment so he could talk to a doctor. After we spoke (with servant), he went to the doctor, and the doctor came, and we arranged everything with him.*” (22 years)

“*We went to the health center, and we talked to those doctors or nurses I mean, they said that they could provide that service. It was 1200 mt (17.1 euros), and they were going to deal with everything. They did not give us the chance to sign a document and follow those procedures.*” (20 years)

## 4. Discussion

The objective of this study was to describe abortion procedures and to explore factors influencing the abortion decision-making process among young women in Maputo and Quelimane.

The study pointed out determinants at the personal, interpersonal and environmental level. Analysing the results, we were confronted with four recurring factors that negatively impacted on the decision-making process: (1) women’s lack of autonomy to make their own decisions regarding the termination of the pregnancy, (2) their general lack of knowledge, (3) the poor availability of local abortion services, and (4) the overpowering influence of providers on the decisions made.

The first factor involves women’s lack of autonomy. In our study, most women indicate that decisions regarding the termination of a pregnancy are mostly taken by others, sometimes against their will. Parents, family members, partners, and providers decide what should happen. As shown in the literature, this lack of autonomy in abortion decision-making is linked to power and gender inequality [34,35,36,37,38]. On the one hand, *power* reflects the degree to which individuals or groups can impose their will on others, with or without the consent of those others [34,37,38]. In this case, the power of the parent/family is observed when they, directly or indirectly, influence their daughters to induce an abortion, for instance by threatening to kick them out of their home. On the other hand, gender inequality is also a factor. This refers to the power imbalance between men and women and is reflected by cases in which the partner makes the decision to terminate the pregnancy [38]. Besides this, the contextual environment of male chauvinism in Mozambique also makes it more socially acceptable for men to reject responsibility for a pregnancy [34,35,37,39,40]. Finally, women’s economic dependence makes them more vulnerable, dependent and subordinated. For economic reasons, women, have no other choice but to obey and follow the family or partner’s decisions. Closely linked with women’s lack of autonomy is their lack of knowledge. Interviewees report that they do not know where abortion services are provided. They are not acquainted with the legal procedures and do not know their sexual rights. This lack of knowledge among women contributes to the high prevalence of pregnancy termination outside of health facilities and not in accordance with legal procedures.

Our participants often report that abortion services are absent at a local level, as has also been pointed out by Ngwena [41]. This is a particular problem in Mozambique. Not all tertiary or quaternary health facilities are authorized to perform abortions. The fact that only some tertiary and quaternary facilities are allowed to do so creates a shortage of abortion centres to cover the demand. In fact, only people with a certain level of education and a sufficiently large social network have access to legal and proper abortion procedures.

Finally, our study shows that providers mostly decide on the location, the methods used and the legality of abortion procedures. Patients are highly dependent on the health providers’ commitment, professionality and accuracy and the selected procedures are not mutually decided by the provider and the patient. Providers often do not refer the client to the reference health facility or do not inform them of the legal procedures, creating a gap between law and practice that stimulates illegal and unsafe procedures. The reasons for this are unclear. It might be due to a lack of knowledge among health providers too, and, perhaps, provider saw here an opportunity to supplement the low salary [42]. Participants who seek help at the health facility they do so contacting the provider in particular, as indication given by someone.

This corroborates with studies conducted by Ngwena [41,43], Doran et al. [44], Pickles [45], Mantshi [46], and Ngwena [47], which pointed out the obstacles related to the availability of services and providers’ attitudes towards safe abortion, although the law grants the population this right [41,43,44,45,46,47]. As Ngwena [41,43] argues, the liberalization of abortion laws is not always put into practice and abortion rights merely exist on paper. Braam’ study [48] therefore highlights the necessity of clarifying and informing women and providers of the current legislation and ensuring that abortion services are available in all circumstances described in the law.

Finally, despite cultural differences between Maputo and Quelimane, the result did not suggest differences between two areas studied regarding factors influencing the decision to terminate and how the abortion is done. However, the Figure 1 suggests that there was trend to have more participants from Maputo reporting abortion episode in her life than Quelimane. This difference maybe be because Maputo is much more multicultural and the people of this city have more access to information that gives them the opportunity to learn about matter of reproductive health including abortion, than Quelimane. So, due to this there is trend decrease the taboo relation to abortion in Maputo than in Quelimane.

These abortion stories illustrate the lack of autonomy in decision-making process given the power and gender inequalities between adults and young women, and also between man and women*.* They also show the lack of knowledge not only on the availability of abortion services at some health facilities, as well as, on the new law on abortion. All these lacks that women have are reinforced by poor availability of abortion services and the fact that the providers we not taking their role to help those women, as it is exposed in the next sections.

This study interviewed young women who had an induced abortion at some point in their lives (15 years up to their age at interview date). As such, it does not provide any information on the factors behind the decisions of those who did not terminate their pregnancy.

The results presented in this paper only reflect the perceptions of the young women who had an induced abortion, not those of their parents or partners. The paper is based on qualitative data that provides insights into factors influencing abortion decision-making. Since the sample included in the study is not representative for the population of young women in Mozambique, the results cannot be generalized.

## 5. Conclusions

Based on the results of the study, we recommend the following measures to improve the abortion decision-making process among young women:

First, strategies should be implemented to increase women's autonomy in decision-making: The study highlighted that gender and power inequalities obstructed young women to make their decision with autonomy. We reiterate the Chandra-Mouli and colleges [49] message. There is a need to address gender and power inequalities. Addressing gender inequality, and promotion of more equitable power relations leads to improved health outcomes. The interventions to promote gender-equitable and power relationships, as well as human rights, need to be central to all future programming and policies [49].

Second, patients and the whole population should be better informed about national abortion laws, the recommended and legal procedures and the location of abortion services, since, despite the decision to terminate pregnancy resulted to the imposition, if they were well informed on that, maybe they could be decide on safe and legal abortion, avoiding double autonomy deprivation. At the same time, providers must be informed about the status of national abortion laws. Additionally, they should be trained in communication skills to promote shared decision-making and patient orientation in abortion counseling. 

Third, the number of health facilities providing abortions services should be increased, particularly in remote areas. 

Finally, health providers should be trained in communication skills to promote shared decision-making and patient orientation in abortion counseling. 

The abortion decision-making by young women is an important topic because it refers the decision made during the transitional period from childhood to adulthood. The decision may have life-long consequences, compromising the individual health, career, psychological well-being, and social acceptance. This paper, on abortion decision-making, calls attention to some attitudes that lead to the illegality of abortion despite it was done at a health facility. 

## Figures and Tables

**Table 1:** Socio-demographic characteristics and abortion procedure.

Characteristics of Respondents	Categories	Median/Number
Age (median, range)	-	21 (min: 17; max: 24)
Age at sexual activity onset (median, range)	-	15.5 (min: 14; max: 18)
Education attainment (number)	Primary school	4
	Secondary School	8
	University	2
Religion (number)	Catholic + Evangelic	13
	Muslim	1
Occupation (number)	Studying	5
	Without occupation	8
	Vendor	1
Abortion procedure		**Number of clients**
Provider characteristics	Skilled	12
	Unskilled	2
Location of abortion	Health facility	7
	Outside of health facility	7
Treatment for abortion	Pills	5
	Aspiration/curettage	8
	Traditional medicine	1
Followed legal procedure	Yes	0
	No	14

**Table 2:** Summary of induced abortion stories. (We changed the table format, please confirm.)

Abortion Stories	Personal	Interpersonal	Environmental
Unwanted pregnancy (5 + 1 *)	Unable to be a mother	Lack of support	The result of rape
	Had a bad past experience		
	Has another child		
	Wanted to study		
	Financial problems		
	Felt depressed		
Abortion although pregnancy is wanted (7)		Partner did not recognize the child	
		Convinced by sister	
		Afraid of being sent away	
		Convinced/forced by mother	
		Partner did not want the child	
		Partner’s behaviour changed	
		Partner was married	
Unskilled provider (2)		Carried out by partner Carried out by mother	
Abortion outside hospital (8)	Unaware of legal obligations	Provider told us to go to his home	Abortion services are not available in the local healthcare settings
	Lack of money		Fear of signing a document
Abortion at home (2)		Mother said that they would kill me at hospital	
		Decided by partner	
Technical procedure		Decided by provider (aspiration, curettage **, pills ***)	
		Husband gave traditional medicine (1)	
Why the legal procedure is not followed in the hospital (6)		Provider did not inform us about it	Information about legal procedures was not available

* The result of rape; ** Seven participants; *** six participants.

## References

[1] Guttmacher Institute In Brief: Facts on the Sexual and Reproductive Health of Adolescent Women in the Developing World Context. http://www.guttmacher.org/pubs/FB-Adolescents-SRH.pdf.

[2] Shah I.H., Åhman E. (2012). Unsafe abortion differentials in 2008 by age and developing country region: High burden among young women. Reprod. Health Matters.

[3] Ustá M.B. O Problema do Aborto Inseguro. Outras Vozes. www.wlsa.org.mz/wp.../11/O-problema-do-aborto-inseguro.pdf.

[4] Hess R.F. (2007). Women’s Stories of Abortion in Southern Gabon, Africa. J. Transcult. Nurs..

[5] Ahman E., Shah I. (2007). Unsafe Abortion : Global and Regional Estimates of the Incidence of Unsafe Abortion and Associated Mortality in 2003.

[6] Ahman E., Shah I. (2004). Unsafe Abortion: Global and Regional Estimates of the Incidence of Unsafe Abortion and Associated Mortality in 2000.

[7] UNFPA (2015). Girlhood, Not Motherhood: Preventing Adolescent Pregnancy.

[8] Ahman E., Shah I. (2011). Unsafe Abortion: Global and Regional Estimates of the Incidence of Unsafe Abortion and Associated Mortality in 2008.

[9] Alhassan A.Y., Abdul-Rahim A., Akaabre P.B. (2016). Knowledge, Awareness and Perceptions of Females on Clandestine Abortion in Kintampo North Municipality, Ghana. Eur. Sci. J..

[10] Gbagbo F.Y., Amo-Adjei J., Laar A. (2015). Decision-Making for Induced Abortion in the Accra Metropolis, Ghana. Afr. J. Reprod. Health.

[11] Olukoya P. (2004). Reducing Maternal Mortality from Unsafe Abortion among Adolescents in Africa. Afr. J. Reprod. Health.

[12] Plummer M.L., Wamoyi J., Nyalali K., Mshana G., Zachayo S., Ross D.A., Wight D. (2008). Aborting and Suspending Pregnancy in Rural Tanzania: An Ethnography of Young People’s Beliefs and Practices. Stud. Fam. Plan..

[13] Lim L., Wong H., Yong E., Singh K. (2012). Profiles of Women Presenting for Abortions in Singapore: Focus on Teenage Abortions and Late Abortions. Eur. J. Obstet. Gynecol. Reprod. Biol..

[14] Kabiru C.W., Ushie B.A., Mutua M.M., Izugbara C.O. (2016). Previous induced abortion among young women seeking abortion-related care in Kenya : A cross-sectional analysis. BMC Pregnancy Childbirth.

[15] Ustá M.B., Mitchell E.M., Gebreselassie H., Brookman-Amissah E., Kwizera A. (2008). Who is Excluded When Abortion Access is Restricted to Twelve Weeks? Evidence from Maputo, Mozambique. Reprod. Health Matters.

[16] Agadjanian V. (1998). “Quasi-Legal” Abortion Services in a Sub-Saharan Setting: Users’ Profile and Motivations. Int. Fam. Plan. Perspect..

[17] Machungo F., Zanconato G., Bergstrom S. (1997). Reproductive Characteristics and Post- Abortion Health Consequences in Women Undergoing Illegal and Legal Abortion in Maputo. Soc. Sci. Med..

[18] Hardy E., Bugalho A., Faúndes A., Duarte G.A., Bique C. (1997). Comparison of Women Having Clandestine and Hospital abortions: Maputo, Mozambique. Reprod. Health Matters.

[19] Assembleia da República Boletim da República: Lei No. 35/2014 de 31 de Dezembro. 14.o Suplemento Imprensa. Maputo, Mocambique; Report No. 105; 2014. http://www.wlsa.orgmz/wp-content/uploads/2014/11/lei-35_2014Codigo_Penal.pdf.

[20] Ministério da Saúde Boletim da República: Diploma Ministerial No. 60/2017 de 20 de Setembro. Maputo, Moçambique; (I). Report No. 147; 2017. www.wlsa.org.mz/wp-content/.../Diploma_ministerial_60-2017.pdf.

[21] Cockrill K., Upadhyay U.D., Turan J., Foster D.G. (2013). The Stigma of Having an Abortion: Development of a Scale and Characteristics of Women Experiencing Abortion Stigma. Perspect. Sex. Reprod. Health.

[22] Kumar A., Hessini L., Mitchell E.M.H. (2009). Conceptualising abortion stigma. Culture Health Sex..

[23] Singh S. (2006). Hospital Admissions Resulting from Unsafe Abortion: Estimates from 13 Developing Countries. Lancet.

[24] Ministério da Saude (MISAU), Instituto Nacional de Estatística (INE) (2013). Inquérito Demográfico e de Saúde 2011.

[25] Associação Mocambiçana Para Desenvolvimento da Família (AMODEFA) (2017). Estatisticas de Serviços Prestados em Saúde Sexual e Reproductiva.

[26] Direcção Nacional de Planificação (2014). Relatório Nacional do Ministério da Saúde.

[27] World Health Organization (1996). Studying Unsafe Abortion: A Practical Guide.

[28] Cleland J., Ingham R., Stone N. (2001). Asking Young People about Sexual and Reproductive Behaviours: Illustrative Core Instruments.

[29] Creswell J.W. (2003). Research Design Qualitative Quantitative and Mixed Methods Approach.

[30] Strauss A., Corbin J. (1998). Basics of Qualitative Research: Techniques and Procedures for Developing Grounded Theory.

[31] Bronfenbrenner U. (1994). Ecological Model of Human Development. International Encyclopedia of Education.

[32] Bronfenbrenner U. (1979). The Ecology of Human Development: Experiments by Nature and Design.

[33] Gerhardt T.E., Silveira D.T. (2009). Métodos de Pesquisa.

[34] John O.L. (2017). Power Dynamics, Gender Relations and Decision-Making Regarding Induced Abortion among University Students in Nigeria. Afr. Popul. Stud..

[35] John O.L. (2016). Sexual Behaviour, Unwanted Pregnancy and Tripartite Levels of Decision-Making Regarding Induced. Afr. J. Psychol. Study Soc. Issues.

[36] Ezeah P., Chinyere A. (2015). Gender Inequality in Reproductive Health Services and Sustainable Development in Nigeria: A Theoretical Analysis. Int. J. Sociol. Anthropol..

[37] Letamo G. (2011). The Influence of Gender Role Attitudes on Risky Sexual Behaviour: Evidence from the 2008 Botswana AIDS Impact Survey III. Afr. Popul. Stud..

[38] Shearer C.L., Hosterman S.J., Gillen M.M., Lefkowitz E.S. (2005). Are Traditional Gender Role Attitudes Associated with Risky Sexual Behavior and Condom-Related Beliefs?. Sex Roles.

[39] Rollins B.C., Bahr S. (1976). A Theory of Power Relationships in Marriage. J. Marriage Fam..

[40] Amaro H. (1995). Love, Sex, and Power: Considering Women’s Realities in HIV Prevention. Am. Psychol..

[41] Ngwena C. (2010). Inscribing Abortion as a Human Right: Significance of the Protocol on the Rights of Women in Africa. Hum. Rights Q..

[42] USAID Avaliação da Corrupção: Moçambique. Relatório Final; December 2005. wwwpdf.usaid.gov/pdf_docs/Pnadg268.pdf.

[43] Ngwena C. Using Human Rights to Combat Unsafe Abortion: What needs to be Done?. www1.chr.up.ac.za/africlaw/charles_ngwenya.pdf.

[44] Doran F., Nancarrow S. (2015). Barriers and facilitators of access to first-trimester abortion services for women in the developed world: A systematic review. J. Fam. Plan. Reprod. Health Care.

[45] Pickles C. (2013). Lived Experiences of the Choice on Termination of Pregnancy Act 92 of 1996: Bridging the Gap for Women in Need. South Afr. J. Hum. Rights.

[46] Mantshi E.T., Laetitia C.R. (2017). “I am all alone”: Factors influencing the provision of termination of pregnancy services in two South African provinces. Glob. Health Action.

[47] Ngwena C. (2000). The Recognition of Access to Health Care as a Human Right in South Africa: Is It Enough?. Health Hum. Rights.

[48] Braam T., Hessini L. (2004). The Power Dynamics Perpetuating Unsafe Abortion in Africa: A Feminist Perspective. Afr. J. Reprod. Health.

[49] Chandra-Mouli V., Svanemyr J., Amin A., Fogstad H., Say L., Girard F., Temmerman M. (2015). Twenty Years After International Conference on Population and Development: Where Are We With Adolescent Sexual and Reproductive Health and Rights?. J. Adolesc. Health.

